# TransFlow: a Snakemake workflow for transmission analysis of *Mycobacterium tuberculosis* whole-genome sequencing data

**DOI:** 10.1093/bioinformatics/btac785

**Published:** 2022-12-05

**Authors:** Junhang Pan, Xiangchen Li, Mingwu Zhang, Yewei Lu, Yelei Zhu, Kunyang Wu, Yiwen Wu, Weixin Wang, Bin Chen, Zhengwei Liu, Xiaomeng Wang, Junshun Gao

**Affiliations:** The Institute of TB Control, Zhejiang Provincial Center for Disease Control and Prevention, Hangzhou, Zhejiang 310051, China; Key Laboratory of Precision Medicine in Diagnosis and Monitoring Research of Zhejiang Province, Hangzhou, Zhejiang 310020, China; The Institute of TB Control, Zhejiang Provincial Center for Disease Control and Prevention, Hangzhou, Zhejiang 310051, China; Key Laboratory of Precision Medicine in Diagnosis and Monitoring Research of Zhejiang Province, Hangzhou, Zhejiang 310020, China; The Institute of TB Control, Zhejiang Provincial Center for Disease Control and Prevention, Hangzhou, Zhejiang 310051, China; The Institute of TB Control, Zhejiang Provincial Center for Disease Control and Prevention, Hangzhou, Zhejiang 310051, China; Department of Medical Oncology, Zhejiang Chinese Medical University, Hangzhou, Zhejiang 310053, China; Key Laboratory of Precision Medicine in Diagnosis and Monitoring Research of Zhejiang Province, Hangzhou, Zhejiang 310020, China; The Institute of TB Control, Zhejiang Provincial Center for Disease Control and Prevention, Hangzhou, Zhejiang 310051, China; The Institute of TB Control, Zhejiang Provincial Center for Disease Control and Prevention, Hangzhou, Zhejiang 310051, China; The Institute of TB Control, Zhejiang Provincial Center for Disease Control and Prevention, Hangzhou, Zhejiang 310051, China; Key Laboratory of Precision Medicine in Diagnosis and Monitoring Research of Zhejiang Province, Hangzhou, Zhejiang 310020, China

## Abstract

**Motivation:**

Whole-genome sequencing (WGS) is increasingly used to aid the understanding of *Mycobacterium tuberculosis* (MTB) transmission. The epidemiological analysis of tuberculosis based on the WGS technique requires a diverse collection of bioinformatics tools. Effectively using these analysis tools in a scalable and reproducible way can be challenging, especially for non-experts.

**Results:**

Here, we present TransFlow (Transmission Workflow), a user-friendly, fast, efficient and comprehensive WGS-based transmission analysis pipeline. TransFlow combines some state-of-the-art tools to take transmission analysis from raw sequencing data, through quality control, sequence alignment and variant calling, into downstream transmission clustering, transmission network reconstruction and transmission risk factor inference, together with summary statistics and data visualization in a summary report. TransFlow relies on Snakemake and Conda to resolve dependencies among consecutive processing steps and can be easily adapted to any computation environment.

**Availability and implementation:**

TransFlow is free available at https://github.com/cvn001/transflow.

**Supplementary information:**

Supplementary data are available at *Bioinformatics* online.

## 1 Introduction

Tuberculosis (TB), caused by *Mycobacterium tuberculosis* complex (MTBC) bacteria, remains a globally severe public health threat, as it causes high mortality induced by a single pathogen (World Health Organization, 2021). Rapid detection of *M.tuberculosis* transmission can offer enhanced opportunities for TB control (Jensen *et al.*, 2005). Genotyping and sequencing methods have revolutionized infectious disease surveillance (Tang *et al.*, 2017). Molecular surveillance combining molecular data with classical epidemiological data allows the investigation of the transmission of disease within the population and the sensitive detection of outbreaks (De Beer *et al.*, 2014; Gavín *et al.*, 2012; Wyllie *et al.*, 2018).

Molecular detection of TB outbreaks is shifted from fingerprinting [mycobacterial interspersed repetitive-unit-variable-number tandem-repeat (MIRU-VNTR)] methods, and sequence-based genotyping assays (multi-locus sequence typing) to next-generation sequencing-based whole-genome sequencing (WGS) in recent years (Struelens and Brisse, 2013). In a retrospective observational TB study in the UK (referred to as UKTB), researchers measured genomic diversity using WGS within community-based MIRU-VNTR-defined clusters and proposed 5 and 12 single nucleotide polymorphisms (SNPs) as potential cutoffs for epidemiological relatedness (Walker *et al.*, 2013). Another population-based, retrospective TB study in Shanghai, China (referred to as CTB), utilized both VNTR and WGS strategies to detect the recent transmission of 324 multidrug-resistant (MDR) tuberculosis strains and demonstrated WGS can measure the heterogeneity of drug-resistant mutations within and between hosts and help to determine the transmission patterns of MDR TB (Yang *et al.*, 2017). Along with the UKTB and CTB studies, multiple studies from around the world have pointed out that the resolution of WGS is superior to that of MIRU-VNTR typing and that epidemiological links can be traced more accurately (Bainomugisa *et al.*, 2021; Bjorn-Mortensen *et al.*, 2016; Folkvardsen *et al.*, 2017; Ford *et al.*, 2012; Jiang *et al.*, 2020).

In the above TB transmission studies based on WGS, the general first step is to define ‘transmission clusters’, sets of cases that are potentially linked by direct transmission (Hatherell *et al.*, 2016). To address this, the most common approach is to use an SNP cutoff-based clustering method which places two cases in the same putative transmission cluster if there are less than a threshold number of SNPs between their sequenced TB genomes (Hatherell *et al.*, 2016). However, it is not yet clear if a single threshold could be used to detect epidemiologically linked cases in all timeframes and contexts (Menardo *et al.*, 2019). Beyond SNP-based clustering, a novel probabilistic approach named TransCluster has been developed (Stimson *et al.*, 2019). In contrast to the SNP cutoff-based clustering, TransCluster clusters sample pairs together if it estimates that there are fewer than a threshold number of transmission events between them, with a given probability. This may outperform the SNP-based method where clock rates are variable and sample collection times are spread out (Stimson *et al.*, 2019).

The second step is inferring the transmission network (‘who infected whom’) of TB from both genetic and epidemiological data (Teunis *et al.*, 2013). In recent years, statistical methods for reconstructing potential transmission links have been rapidly developing, such as SeqTrack, TransPhylo, Outbreaker2 and SCOTTI (Campbell *et al.*, 2018; Didelot *et al.*, 2017; Jombart *et al.*, 2011). Two of them (SeqTrack and TransPhylo) were used to analyze MTB outbreaks before (Ayabina *et al.*, 2018; Didelot *et al.*, 2017; Guerra-Assunção *et al.*, 2015). These methods utilize genomic data, either directly as a multiple sequence alignment (Outbreaker2 and SeqTrack), or indirectly from a timed phylogenetic tree (TransPhylo and SCOTTI), as well as sampling dates. SeqTrack is the fastest tool due to the simplicity compared to other models which employ a Bayesian framework that have to run over millions of Markov Chain Monte Carlo iterations for chain convergence. Outbreak2 and TransPhylo have been developed to account for the complex epidemiology, including handling within-host evolution and non-complete outbreak sampling. Notably, SeqTrack and Outbreak2 can use spatial or contact-tracing data to improve the transmission network reconstruction, respectively. Additionally, for a central goal of TB control, it is of importance to figure out transmission risk factors to identify highly contagious TB patients (Meehan *et al.*, 2019).

Despite decreasing costs to integrate sequencing technologies into routine TB molecular surveillance, many laboratories still lack the computational resources and specialized staff required for analyzing and managing sequencing data (Meehan *et al.*, 2019). There are several open-source or commercially available bioinformatics pipelines and websites automating MTBC sequencing data manipulation and analysis in a single step, such as TB-Profiler (Phelan *et al.*, 2019), Mykrobe (Hunt *et al.*, 2019), MTBSeq (Kohl *et al.*, 2018) and SAM-TB (Yang *et al.*, 2022). All of them provide the functions of anti-TB drug-resistance prediction and MTB lineage classification from sequencing reads. Besides, both MTBseq and SAM-TB provide the analysis of genetic relationships, and SAM-TB further integrates the identification of non-tuberculous mycobacteria species. However, there remains a lack of a standardized and validated data analysis workflow primarily for the identification of recent transmission chains and their direction (Jajou *et al.*, 2019; Meehan *et al.*, 2019).

In this article, we present a novel workflow named TransFlow which uses a modern computational workflow management system, Snakemake (Koster and Rahmann, 2012), to combine many of the state-of-the-art tools currently employed in WGS-based MTBC transmission analysis into a single, fast, easy-to-use pipeline. TransFlow is scalable since it can be run on either computing servers with many cores (which enable parallel computing) or on a personal computer with limited computing resources. TransFlow is also flexible and configurable: it adopts both SNP-based and transmission-based methods for transmission clustering and can further incorporate other epidemiological data for molecular surveillance based on the user’s settings and inputs. We apply this workflow to two real WGS datasets from the CTB and UKTB studies to show its functions and performance. Meanwhile, we provide documentation, example data, outputs and a sample report on the official GitHub repository to facilitate rapid evaluation and adoption of our workflow.

## 2 Implementation

**Fig. 1.**
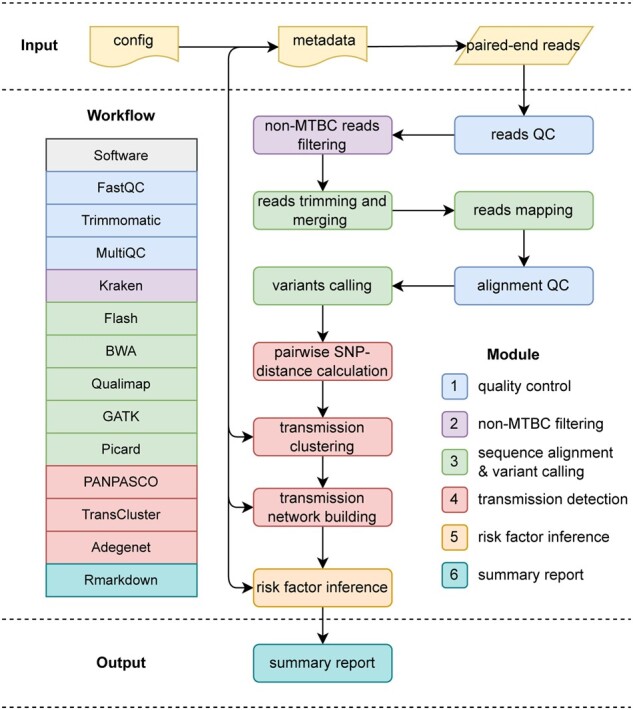
Overview of the full workflow and tools performed by TransFlow (Transmission Workflow). The different modules of the pipeline are broken down by colors

The analysis steps of TransFlow are expressed in terms of ‘rules’ connecting input files to output files as part of the overall workflow (Fig. 1). Upon execution, Snakemake infers the combination of rules necessary to achieve a ‘target’ or specific output, in our case, the final summary report (referred to as the report file). The necessary steps will be run in an optimized manner depending on the computational environment.

TransFlow runs from a single configuration file (referred to as the config file), where users list their pair-end sequences FASTQ files and certain parameters about the analysis in human-readable YAML format (Supplementary Section S1). It also takes a single TSV format file (Supplementary Section S2) with metadata of samples including at least sample ids and collection dates (referred to as the metadata file) as input. The overall TransFlow framework is comprised of five distinct and coherent analysis modules: (i) quality control, (ii) MTBC filtering, (iii) sequence alignment and variant calling, (iv) transmission detection and (v) transmission risk factor inference. For detailed information including software requirements, default parameters, usages and descriptions, please see Supplementary Section S3. A Shell script is provided to automatically run a complete analysis with all modules combined. In addition, each module can be run independently, so that users can obtain satisfactory results by adjusting relevant parameters, such as manually filtering out low-quality samples, trying different transmission detection methods or thresholds, etc. A significant and unique advantage of TransFlow is that its underlying framework enables easy and efficient rerunning of analyses. Unless the relevant input files have been changed, upstream steps of the pipeline will not be re-executed. Users can easily re-execute steps if errors occur, or the data and parameters need to be adjusted.

TransFlow is fully open source and implemented in both Python and R programming languages. It uses the Conda environment manager (Anaconda,Inc., 2020) for extensive control of external tools, including versioning of configurations and environments, provenance capabilities and scalability on high-performance computing clusters. The common parameter settings for different modules were predefined, and some can be straightforwardly customized to meet users’ specific needs. Complete usage and user options are outlined in the TransFlow repository. In addition, we provide a toy example dataset including FASTQ and metadata files for processing the whole workflow. For this purpose, short reads were simulated with NEAT (see Supplementary Section S3 for all simulation details) (Stephens *et al.*, 2016).

## 3 Results

To illustrate the utility of TransFlow, we applied it to a real dataset from the CTB study (SRA accession: SRP058221) (Yang *et al.*, 2017). This study collected a total of 324 MTB isolates from MDR TB patients. The authors first screened 125 samples by VNTR genotyping then successfully performed WGS in 122 of them. The epidemiological data were obtained from the authors (Supplementary Table S1).

### 3.1 MTBC filtering

It is important to filter out samples that may have been significantly contaminated by foreign DNA during sample preparation. The paired-end reads of each sample are classified through Kraken (Wood and Salzberg, 2014) against a pre-built database (MiniKraken DB_8GB, October 18, 2017: https://ccb.jhu.edu/software/kraken/) containing all of the complete genomes of bacteria, archaea, virus, protozoa, plasmids and fungi in RefSeq (Haft *et al.*, 2018). A custom Python script is used to calculate the proportion of reads that are taxonomically classified under the MTBC for each sample and implement a defined threshold (default 90%) (Ezewudo *et al.*, 2018; Vargas *et al.*, 2021). A sample will be dropped if it has less than this threshold of reads aligned to the MTBC (Supplementary Table S2). Besides, TransFlow provides an option that allows users to lower the MTBC screening threshold and filter out non-MTBC reads with Kraken in the meantime so that contaminated samples can still be reliably processed.

### 3.2 Quality control of raw reads and alignments

**Fig. 2.**
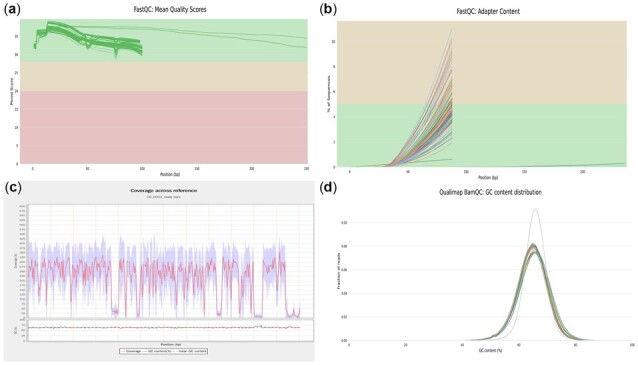
Quality control of raw reads and alignment. (**A**) Mean quality value across each base position in the read. (**B**) Cumulative percentage count of the proportion of adapter sequences. (**C**) Alignment coverage and GC content across reference genome. (**D**) Distribution of GC content of mapped reads of all samples

FastQC (https://www.bioinformatics.babraham.ac.uk/projects/fastqc/) is adopted to check the quality of the sequencing reads and produces a report for each FASTQ file. TransFlow uses MultiQC (Ewels, 2016) to summarize all the reports and merge them into an integrated report, as shown in Figure 2A and B. Users can then check the report and set up the parameters for Trimmomatic (Bolger *et al.*, 2014), such as ILLUMINACLIP, SLIDINGWINDOW and MINLEN. The raw reads quality of the CTB dataset is not good enough since there is a large number of adapter sequences present in some FASTQ files, and adapter trimming is therefore performed.

After the step of alignment to the reference genome, the intermediate output BAM files will be provided to Qualimap2 (Okonechnikov *et al.*, 2015) to evaluate the alignment quality. Figure 2C shows an example plot from Qualimap2. MultiQC is then used to generate a statistics figure on the GC content using the output of feature counting (Fig. 2D).

### 3.3 Pan-genome-based pairwise SNP distances

**Fig. 3.**
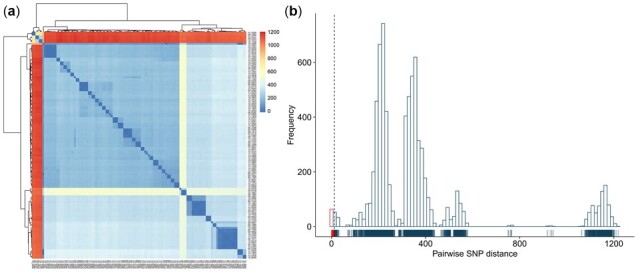
Pan-genome-based pairwise SNP distances between samples. (**A**) Heatmap representation of the pairwise SNP distances shows genetic similarities and differences among all samples. (**B**) Histogram represents the distribution of all pairwise SNP distances. The peaks represent the genetic differences between major lineages. The dashed line denotes a cutoff of 12 SNPs. Rug lines representing individual pairs are shown at the bottom

To overcome the bias of the lineage-specific reference genomes, TransFlow adopts the PANPASCO pipeline (Jandrasits *et al.*, 2019) to perform pairwise SNP distance calculation, which uses a computational pan-genome incorporating 146 MTBC complete genomes representing the main lineages 1–4. Furthermore, BWA (Li and Durbin, 2009), SAMTools (Li *et al.*, 2009) and GATK (DePristo *et al.*, 2011) are utilized in TransFlow for sequence alignment and variant detection, separately. Additionally, SNPs annotated in regions difficult to map such as repetitive sequences and PPE/PE-PGRS genes of the reference pan-genome are excluded (Meehan *et al.*, 2019). PANPASCO generates a TSV file containing a symmetric matrix of pairwise SNP distances among all samples. After that, TransFlow first outputs a clustered heatmap to display this matrix (Fig. 3A). Secondly, TransFlow draws a histogram to display the distribution of all genetic distances in which the distances ranging from 0 to 12 are highlighted. Both figures show primary evidence of putative recent transmissions (Fig. 3B).

### 3.4 Transmission detection

**Fig. 4.**
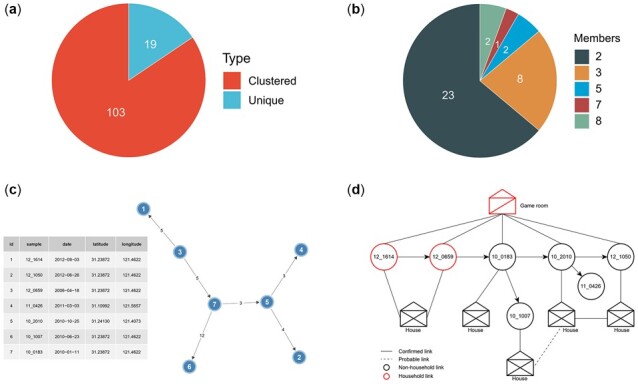
Transmission clustering and network reconstruction. (**A**) Statistics of clustered and unique samples. (**B**) Statistics of members of all transmission clusters. (**C**) Inferred transmission network of Cluster 2 based on pairwise SNP distances, sampling dates and geographic coordinates. Each node represents a clustered strain. The number of SNPs that separate the different strains within and between clusters is specified. Arrows indicate the potential direction of transmission within clusters. The detailed information is shown in the right table. (**D**) Putative transmission scenario inferred from both the inferred transmission network and epidemiological links [based on Yang and colleagues’ study (Yang *et al.*, 2017)]

After pairwise SNP distance calculation is completed, TransFlow makes use of the R package TransCluster (Stimson *et al.*, 2019) to perform transmission clustering. TransCluster provides two different clustering methods, SNP-based and transmission-based methods to infer the samples potentially linked by recent transmission. For the SNP-based method, two samples are in the same transmission cluster if their SNP distance is less than or equal to a fixed cutoff. The SNP-based method only considers the SNP distances, while the transmission-based method further takes into account the priors of sampling dates, clock rate and transmission processes. The transmission-based method is to cluster sample pairs together if the number of estimated transmission events between them is lower than a threshold number, at a given probability of 80%. The transmission rate is the rate at which intermediate cases occur in the total time elapsed between the most recent common ancestor of two sampled hosts and sampling events. The molecular clock rate of MTB is estimated from 0.04 to 2.2 SNPs/genome/year, with substantial differences between lineages (Menardo *et al.*, 2019). The transmission clusters were to portray not only recent direct transmission events within the study population but also earlier transmission events that were connected by unsampled contacts (Stimson *et al.*, 2019). This step outputs a TSV file containing both the clustering results of all samples and the cluster ids of the clustered samples (Supplementary Table S3). The clusters are sorted by the number of their members. Furthermore, TransFlow generates visualizations including two pie charts to show the statistics of clustered samples (Fig. 4A) and the members of all clusters (Fig. 4B), separately.

Next, the transmission network reconstruction is performed using the SeqTrack algorithm (Jombart *et al.*, 2011) from R package Adegenet (Jombart, 2008) on the clusters including at least three samples for ensuing analyses. Besides the SNP distances and sampling dates, users can further input the geographic coordinates of samples to represent their spatial connectivity and improve the local transmission inference. These inputs are then handled by the ggnet2 function from the R package GGally (Shannon *et al.*, 2003) to generate network visualizations and corresponding nodes and link files (Fig. 4C). It is worth noting that these files can be directly imported into Cytoscape (Shannon *et al.*, 2003) to manually modify the transmission network.

### 3.5 Transmission risk factor inference

**Fig. 5.**
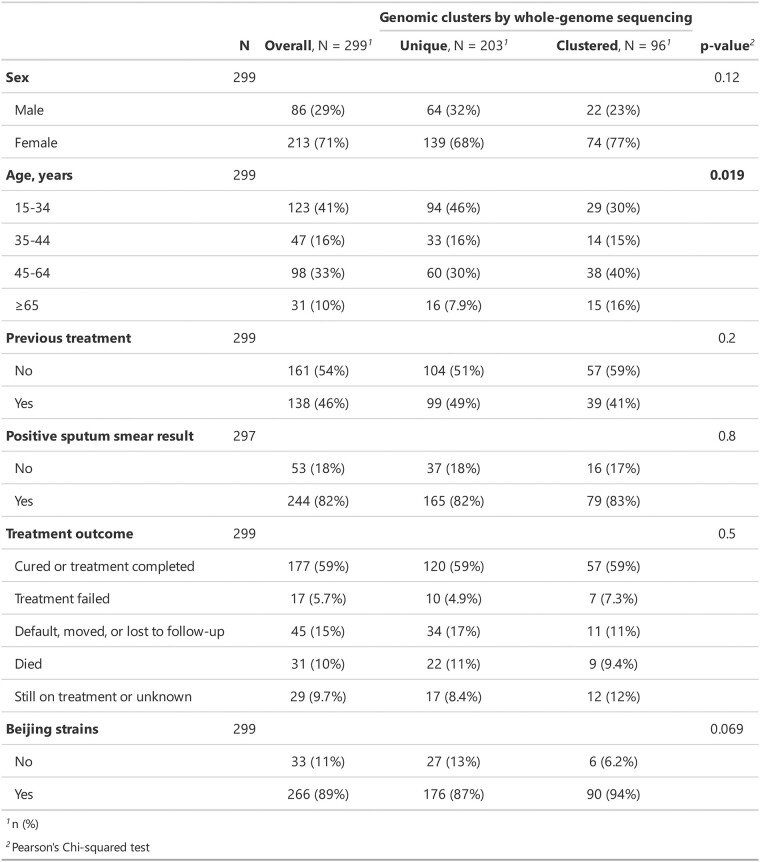
Univariable analysis of risk factors for TB transmission. Bold values denote statistical significance at the *P*-value < 0.05 level

TransFlow further provides a function for inferring epidemiological risk factors related to transmission. Users are required to provide all epidemiological characteristics data to be detected in the metadata file, such as age, gender, place of residence, previous TB treatment history and status of Diabetes or HIV infection. TransFlow uses the R package gtsummary to perform univariate regression analysis on the epidemiological characteristics specified in the config file with transmission clustering (Sjoberg *et al.*, 2021). It automatically detects continuous, categorical and dichotomous variables in the data set, performs appropriate descriptive statistics and also includes the amount of missingness in each variable (details are in Supplementary Section S3). Finally, it generates a publication-ready analytical and summary table (Fig. 5).

### 3.6 Creation of summary reports

The results of the TransFlow are presented in a user-friendly interactive HTML report which is generated using a custom R markdown script and rendered with the R package knitr (Xie, 2018). The report contains summary statistics, visualization plots and descriptions of the pairwise SNP distances, transmission clusters, transmission networks and risk factor inference (Supplementary Section S4).

### 3.7 Real datasets results

For the CTB dataset, we test the transmission-based method with a clock rate of 1.5 SNPs/genome/year, a transmission rate of 2.0 and a transmission threshold of 19. In consideration of the reports of higher mutation rates of MDR strains (Borrell and Gagneux, 2009; de Steenwinkel *et al.*, 2012), the clock rate selected is larger than the typical rate for TB but within the range of recently reported mutation rates of Beijing-family strains (Menardo *et al.*, 2019). The transmission rate is also within the range of potential transmission rates from the original TransCluster paper (Stimson *et al.*, 2019). According to a recent TB research in Malaysia, we also selected the transmission threshold of 19 (Bainomugisa *et al.*, 2021). A total of 103 (84%) of 122 sequenced strains (Fig. 4A) in 36 putative transmission clusters are identified (Fig. 4B), which is almost the same as the results in the original study of the CTB dataset (103 [84%] in 38 clusters).

For example, Figure 4C shows the reconstructed transmission network of Cluster 2, which is the same as Cluster 9 in the original CTB study and supplements the putative transmission traces as well. We can further manually integrate the inferred transmission network and epidemiological links from the original paper to recover a putative transmission scenario as shown in Figure 4D. The putative index case was a husband (12_1614) who then transmitted MTB to his wife (12_0659). Afterward, transmission events occurred in the game room of a residential complex which resulted in infection to other three patients (10_0183, 10_2010 and 12_1050). Besides, we can identify two patients without any epidemiological link to the game room (10_1007 and 11_0426) who are linked to patients 10_0183 and 10_2010, separately in a transmission chain.

To identify risk factors associated with the transmission, 177 cases identified as unique by the VNTR genotyping in the previous CTB study population are also incorporated (Supplementary Table S1). Differences in six epidemiological characteristics (age, sex, treatment history, sputum smear result, treatment outcome and Beijing lineage) between clustered and unique cases are assessed among a total of 299 cases with available epidemiological investigation results. Consistent with the original paper, the results indicated that age is a putative risk factor for the transmission of multidrug-resistant TB (Fig. 5), which means a patient being 45 years or older is more likely to be in a transmission cluster of MDR tuberculosis than other patients.

**Fig. 6.**
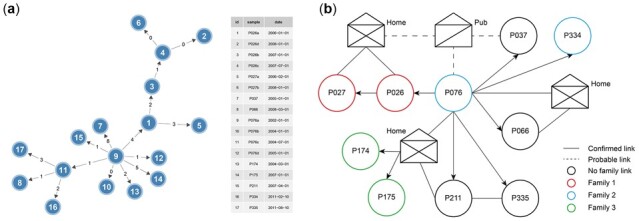
Transmission network (**A**) reconstructed with TransFlow between samples of the UKTB dataset. (**B**) Putative transmission scenario inferred from both the inferred transmission network and epidemiological links based on the original UKTB study (Walker *et al.*, 2013)

We also evaluated TransFlow for the second dataset, UKTB and focused on a transmission cluster described in detail (Supplementary Table S4). This community cluster (Cluster 7, original paper) was initially defined by the shared MIRU-VNTR profile of the samples and includes 17 sequenced isolates of ten TB patients with one central, treatment non-compliant individual (Walker *et al.*, 2013). Through epidemiological investigation, the authors found that the SNP differences between all strains with known or possible epidemiological links in this cluster did not exceed 12 SNPs. Accordingly, using TransFlow with the same cutoff of 12 SNPs, we identified the same transmission cluster and reconstructed the transmission network (Fig. 6). The transmission direction speculated by TransFlow shows the putative index case was a super-spreader in this transmission cluster. P076 transmitted MTB to nearly all other patients including one family member (P334). Transmission events might occur in a pub which resulted in infection to two other patients (P037 and P026) and then P026 transmitted MTB to family member P027 at home. Also, we can identify that P066 was infected by P076 through a household epidemiological link. There was another household epidemiological link from P076 to four other patients (P174, P175, P211, and P335), among whom P174 and P175 were from the same family.

### 3.8 Performance testing

To demonstrate TransFlow’s performance, we first measured the run time and maximum memory usage via the Linux time command for a subset of 50 samples from the CTB dataset when invoked with a certain thread count, in the range from 8 to 56, respectively. Supplementary Figure S1A shows that an improved performance is observed with 32 threads but beyond that point the performance improvements diminish greatly. The memory usage is consistent with increasing threads at about 6 Gb (Supplementary Fig. S1B). In addition, Supplementary Figure S1C shows that the run time is proportional to the increase in sample size from the CTB dataset. The memory usage only increased from 30 to 60 samples and eventually stabilize at about 7 Gb (Supplementary Fig. S1D). All tests were performed in a server with double Xeon Platinum 8268 2.90 GHz CPU (total 48 cores and total 96 threads) and 1Tb shared RAM 2133 MHz.

## 4 Discussion

TransFlow is designed following three core concepts that permeate throughout the design of the pipeline. First, it is designed with visualization of results as a key principle to generate output encapsulating important analysis results in informative, publication-quality figures. Secondly, TransFlow is developed based on Snakemake to acquire both efficiency and customizability. Lastly, we aim to ensure that TransFlow could be installed and used by anyone, even those with limited bioinformatics experience. Accordingly, the installation of TransFlow requires minimal user input, and the operation can be launched by a single terminal command with inputs generated by any text or table editor.

### 4.1 Utilization of state-of-the-art tools

Bioinformatics technologies for WGS-based tuberculosis molecular epidemiology are still in fast development. However, their applications are debated regarding both the selection of reference genome and the threshold of recent transmission. To overcome these challenges, TransFlow adopts two state-of-the-art tools, PANPASCO and TransCluster. PANPASCO utilizes a pan-genome with the representation of the four main lineages 1–4 and a pairwise distance method to avoid the genetic distance calculation bias (Jandrasits *et al.*, 2019). TransCluster is a novel transmission cluster identification tool, which brings sampling time, SNP distance, transmission rate, and molecular clock rate into its transmission probability model to improve the recognition rate of transmission clusters and the flexibility of samples (Stimson *et al.*, 2019). By taking advantage of both tools, TransFlow identifies two credible clusters (Clusters 1 and 4) which modifies three clusters in the original CTB study (details are described in Supplementary Section S5).

### 4.2 Visualization of analyses results

TransFlow outputs figures or tables for all analyses that allow users to rapidly understand and utilize the analysis results. The most important visualizations are all compiled into a single summary report file, which highlights the main features of the analysis while explaining each of the individual processes needed to create the figure. All the figures are output in both PDF and PNG format to facilitate the publication.

### 4.3 Snakemake as a framework

TransFlow is built upon Snakemake (Koster and Rahmann, 2012), a scalable workflow engine that helps to manage workflows easily. It divides the whole workflow into rules with each rule accomplishing one step of the workflow. The input of one rule is the output from the rule corresponding to the previous step, making the data flow easy to track. TransFlow organizes the rules carrying out a big step of the workflow together in one snakefile. All the modules share a common config file and are then integrated into the main Shell script. Users can call this script to perform an end-to-end analysis or run each module step by step. It is particularly useful when users want to try different parameters, e.g. different clustering methods. Additionally, Snakemake infers which rules are independent of each other and can be run in parallel, thus reducing idle CPU time to speed up workflow completions.

TransFlow is highly modular and open source, thus it allows users to switch tools utilized in the workflow. Following steps should be performed, for example, to switch the sequencing reads alignment program from the default BWA to Bowtie2. First, add Bowtie2 information to the YAML file of Conda environment. Next, modify some contents of the Snakemake rule which uses BWA for reads mapping, including the format of output files and shell commands, to meet the requirements of Bowtie2. Also, modifications are needed correspondingly in other rules where these output files exist as input files.

### 4.4 Ease of use

The documentation for installing, deploying and using TransFlow is provided online. It is worth noting that TransFlow is designed to use the package manager Conda (Anaconda,Inc., 2020) and the Bioconda (The Bioconda Team *et al.*, 2018) channel. This allows users to download and install the dozens of bioinformatics tools and packages that go into TransFlow with a single command. All applications and algorithms incorporated into TransFlow can be fine-tuned in the accompanying configuration file, with each option having a detailed description and recommend default setting. Setting up a metadata file for TransFlow requires basic usage of the terminal and software such as Excel to edit a TSV file, both of which involve very simple commands.

## 5 Conclusions

We present a new WGS-based TB transmission analysis pipeline TransFlow, which is fast, efficient, customizable and easy-to-use, enabling it to be an effective and modern tool for researchers. The complete workflow starts with quality control of the raw reads and MTBC filtering. It goes through several steps including optional trimming, pan-genome reference alignment, variant calling, pairwise SNP distances calculation, transmission clustering, transmission network reconstruction and risk factor inference. A detailed summary report is generated in the end to incorporate all results from previous analysis steps.

We will regularly add more novel workflows which consist of newly developed tools as anything new emerges. We welcome all the feedback from users regarding our pipeline and are always waiting at some point to improve and update the modules to meet the specific demands from them and hope to assist in making full use of the merit of WGS technology as it goes.

## Supplementary Material

btac785_Supplementary_Data

## Data Availability

The code of version 1.0 reported in this article is archived at Zenodo.org: https://doi.org/10.5281/zenodo.703965. Up-to-date code and new releases will be made available on GitHub, together with information on running the workflow locally: https://github.com/cvn001/transflow.
