# Supplementary figures and images for: TransFlow: a Snakemake workflow for transmission analysis of Mycobacterium tuberculosis whole-genome sequencing data
Source: Bioinformatics. 2022 Dec 5;39(1):btac785. doi: 10.1093/bioinformatics/btac785 (PMC9825751; doi:10.1093/bioinformatics/btac785)

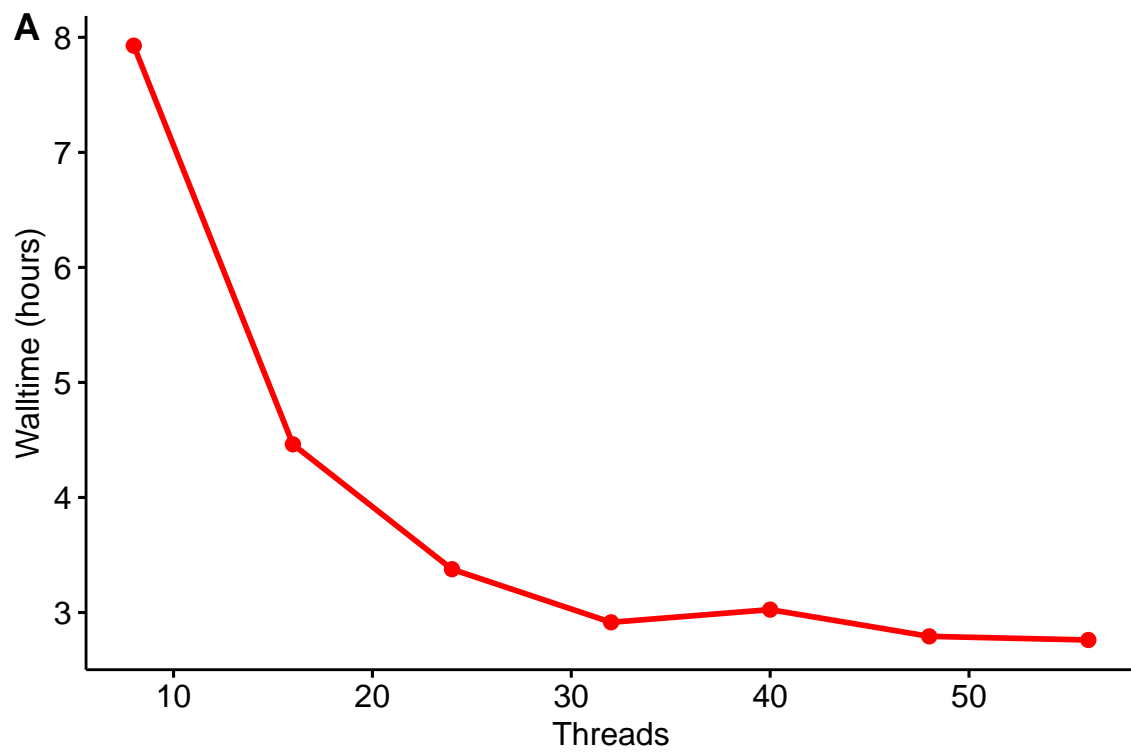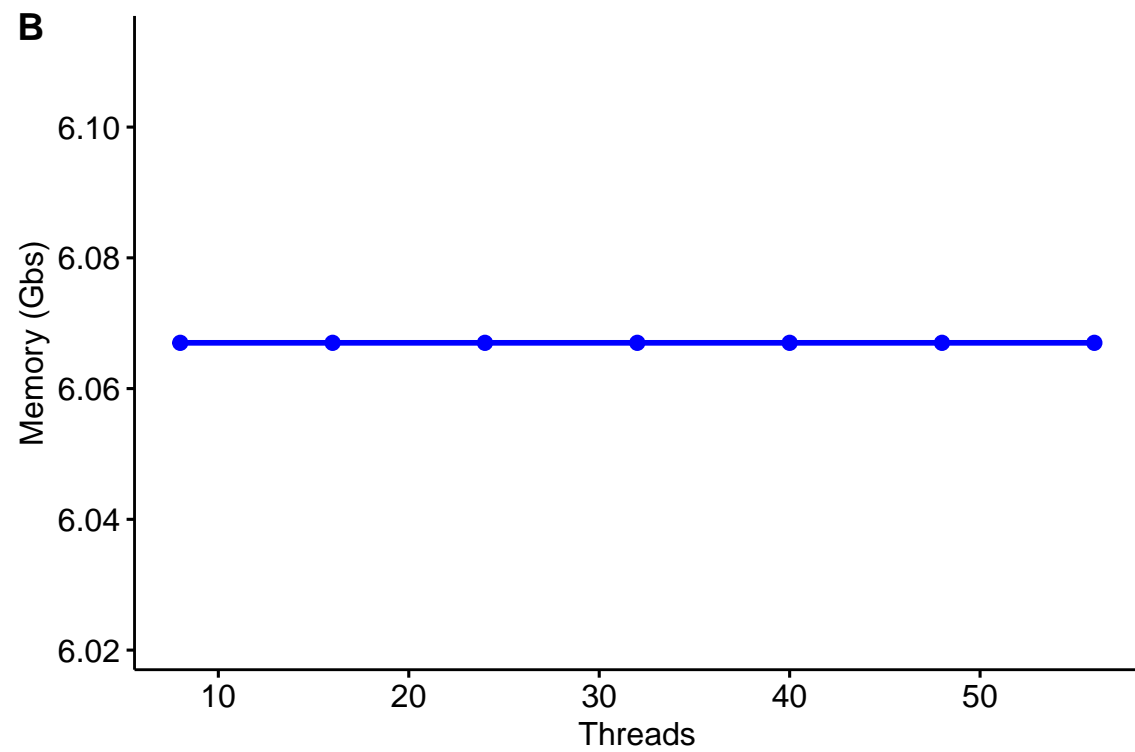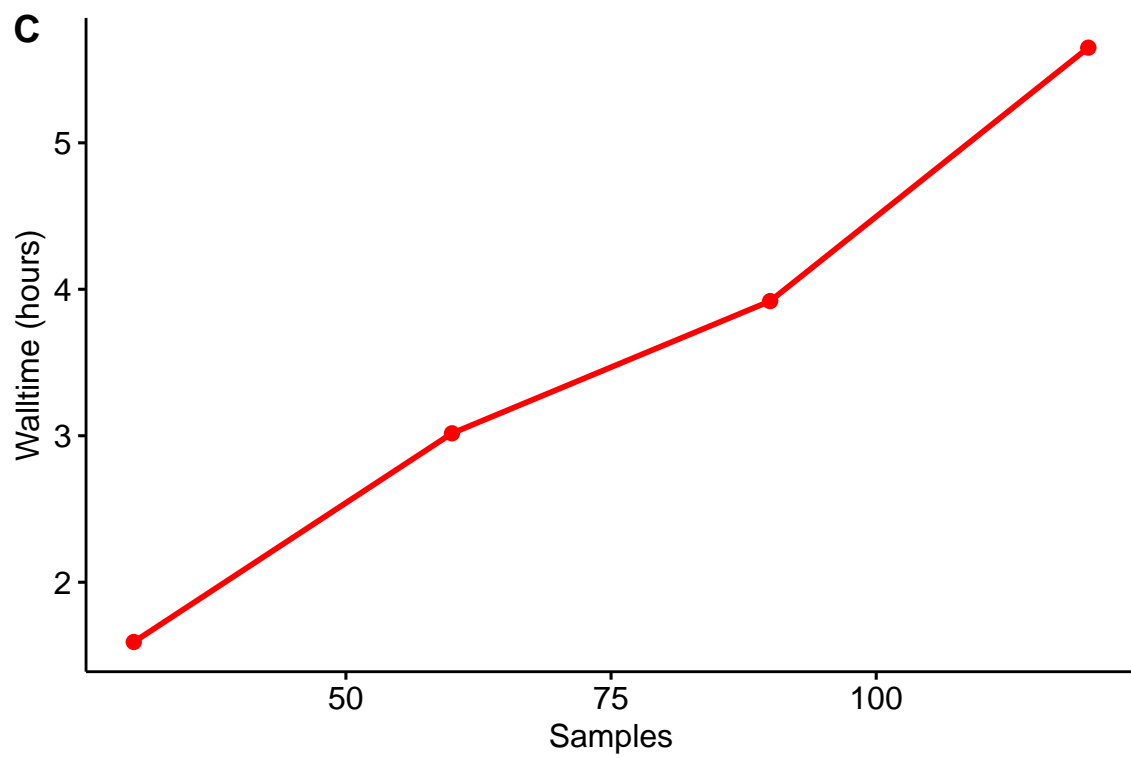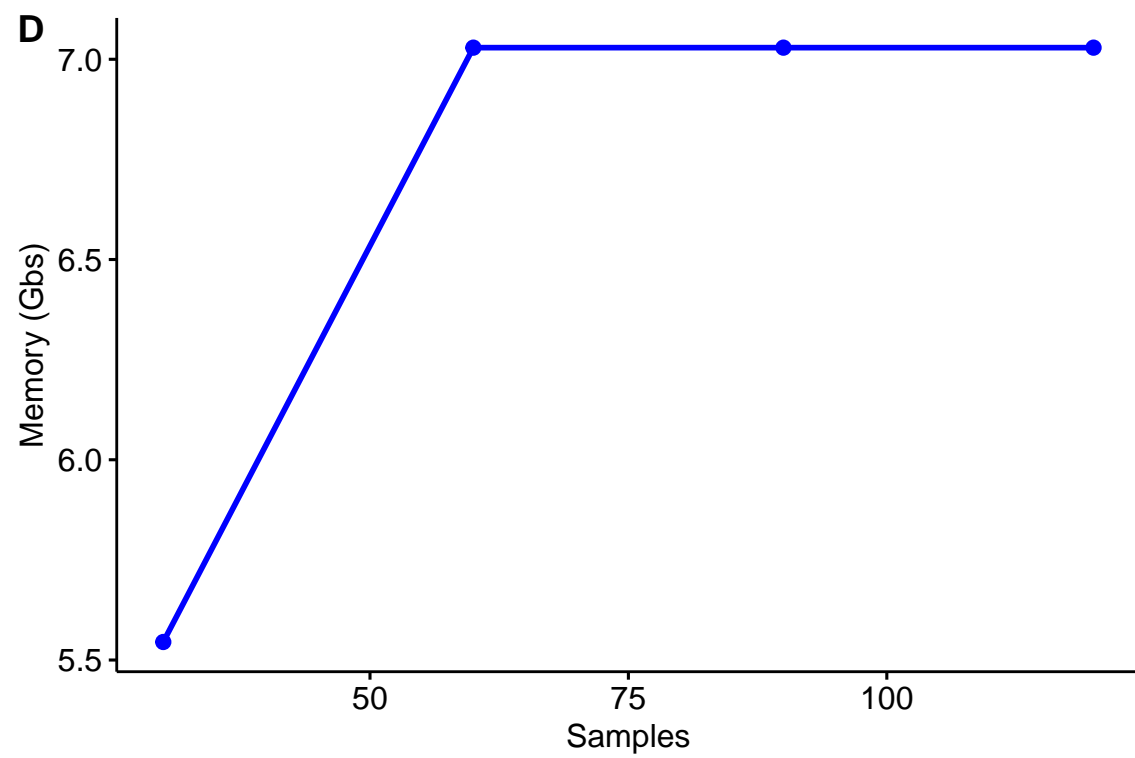

Supplement: btac785_Supplementary_Data [file btac785_supplementary_data.zip › Supplementary_Figure_S1.pdf]
